# Stagnant Water

**Published:** 1873-06

**Authors:** 


### STAGNANT WATER.

Out of one hundred and forty families supplied with milk from a dairy at Islington, England, seventy—just one half—were attacked with typhoid fever, and thirty persons died. The cows were supplied with water from a wooden tank, in a state of decay. None were attacked who did not use the milk. Stagnant water slowly poisons man and beast; hence cattle should be supplied with fresh rain water or from running streams, although they may sometimes seem to prefer drinking from ponds. No doubt many families have suffered from allowing cattle to drink out of pools and standing water. Stagnant water in warm weather teems with living things, exceedingly minute, and if a cow drinks it they will find their way alive into the milk, and if such milk is watered, it will abound with those living creatures. No unhealthful results may be noticed from drinking milk diluted with pure water, but if the milkman supplies himself from a stagnant pool by the way-side, the family are liable to various forms of disease, including maladies resulting from worms living within, unless all milk purchased is boiled before being used.

